# Nonlinear responses of ecosystem carbon fluxes to precipitation change in a semiarid grassland

**DOI:** 10.3389/fpls.2025.1519879

**Published:** 2025-02-06

**Authors:** Hua Chai, Jianying Ma, Jinwei Zhang, Junqin Li, Bo Meng, Chengliang Wang, Duofeng Pan, Jie Li, Wei Sun, Xuhui Zhou

**Affiliations:** ^1^ Key Laboratory of Sustainable Forest Ecosystem Management-Ministry of Education, School of Forestry, School of Ecology, Northeast Forestry University, Harbin, China; ^2^ Institute of Grassland Science, Key Laboratory of Vegetation Ecology of the Ministry of Education, Jilin Songnen Grassland Ecosystem National Observation and Research Station, Northeast Normal University, Changchun, China; ^3^ Key Laboratory of Geographical Processes and Ecological Security in Changbai Mountains, Ministry of Education, School of Geographical Sciences, Northeast Normal University, Changchun, China; ^4^ Department of Grassland Science, College of Animal Science and Technology, Northeast Agricultural University, Harbin, China; ^5^ Institute of Forage and Grassland Sciences, Heilongjiang Academy of Agricultural Sciences, Harbin, Heilongjiang, China

**Keywords:** ecosystem carbon fluxes, altered precipitation, nonlinear responses, precipitation gradient, semiarid grasslands

## Abstract

Carbon (C) fluxes in semiarid grasslands subject to precipitation variability play a critical role in the terrestrial C cycle. However, how ecosystem C fluxes respond to variability in precipitation (both decreases and increases precipitation along a gradient) remains unclear. In this study, we conducted a three-year field experiment in a semiarid grassland, with six precipitation treatments (precipitation decreased by 70%, 50%, and 30% [P–70%, P–50%, and P–30%], natural precipitation [P+0%], and precipitation increased by 30% and 50% [P+30% and P+50%]) to examine how variations in precipitation influence ecosystem C fluxes, specifically focusing on gross ecosystem productivity (GEP), ecosystem respiration (ER), and net ecosystem CO_2_ exchange (NEE). We found that both decreased and increased precipitation significantly altered the GEP (from –26% to 14%), but only decreased precipitation significantly reduced the ER and NEE (from 1% to 31%), relative to their values during natural precipitation. This suggests that ecosystem C fluxes are more sensitive to decreased precipitation, and respond nonlinearly to the precipitation gradient. Furthermore, structural equation modeling indicated that the soil water content was the primary controlling factor driving changes in ecosystem C fluxes. Our research underscores the nonlinear response of ecosystem C fluxes to changes in precipitation within semiarid ecosystems, particularly their sensitivity to extreme drought. Considering this nonlinear response, it is crucial to improve dynamic models of the C cycle and predict ecosystem responses to precipitation variability.

## 1 Introduction

Global changes, marked by rising temperatures and increasing atmospheric CO_2_ concentrations, are reshaping the precipitation patterns that regulate terrestrial ecosystems (Sage, 2020). These transformations of precipitation patterns are reflected in both the increased interannual variability of precipitation and shifts in the seasonal distribution of precipitation events (IPCC, 2013; Knapp et al., 2015). Concurrently, climate models predict a rise in the occurrence and intensity of extreme precipitation events (IPCC, 2013). As a vital force driving ecosystem processes, precipitation changes intricately affect the physiological metabolism of plants, as well as the physical, chemical, and biological processes in the soil, ultimately redefining the carbon (C) cycle in terrestrial ecosystems and their complex feedbacks with climate change (Beier et al., 2012; Chen et al., 2016). Semiarid grasslands account for a significant portion of the world’s grassland area (Post et al., 2021). Due to water limitation, the structure, processes, and functions of semiarid grasslands are highly sensitive to changes in precipitation (Liu et al., 2021). Consequently, the effects of precipitation change on grasslands are more pronounced than the individual or combined effects of increased CO_2_ concentrations and rising temperatures (Song et al., 2019). Therefore, knowing how C fluxes within grassland ecosystems respond to precipitation change is essential for understanding of the global C cycle during climate change.

Precipitation change is the most significant factor shaping C fluxes within grassland ecosystems (Wang et al., 2021b). Regardless of how precipitation patterns change, they invariably influence grassland C fluxes by transforming plant species composition, growth dynamics, and soil water content (SWC) (Morales-Rincon et al., 2021; Wang et al., 2021b; Zhang et al., 2019). However, despite extensive research, our understanding of the intricate relationship between precipitation changes and grassland C fluxes remains incompletely. Some studies have suggested that increased precipitation enriches SWC, elevates plant photosynthesis, fosters growth, and ultimately enhances the net ecosystem CO_2_ exchange (NEE) (Parton et al., 2012). In contrast, drought conditions suppress plant growth, limit net primary productivity, and decrease NEE (Scott et al., 2009). Furthermore, C fluxes in humid grassland ecosystems are insensitive to alternating wet and dry conditions (Jaksic et al., 2006). In addition, numerous *in situ* experiments and modeling studies have revealed that ecosystem C fluxes have both linear responses and nonlinear responses, with thresholds, to changes in precipitation (Huxman et al., 2004; Morales-Rincon et al., 2021; Zhang et al., 2019). These divergent response patterns amplify the uncertainty in predicting how precipitation changes affect C fluxes within grassland ecosystems.

Most studies exploring how changes in precipitation impact C fluxes in grasslands within arid regions rely on interannual and seasonal precipitation variability. Typically, these investigations infer ecosystem responses to precipitation change based on the natural variation in precipitation. However, the effects of other confounding factors, such as temperature, cannot be ruled out. Environmental variables beyond precipitation can shift across time and space, potentially significantly reshaping ecosystem C fluxes (Song et al., 2019). In recent years, field experiments simulating precipitation changes through *in situ* treatments, such as increased or decreased precipitation, have been increasingly used (Wang et al., 2021b). Previous research has shown that, under drought conditions, gross ecosystem productivity (GEP) is often more sensitive than ecosystem respiration (ER), culminating in a suppression of NEE in the face of diminished precipitation (Wu et al., 2011; Zhang et al., 2017). However, other studies have revealed a different trend: in arid and semiarid ecosystems, surges in GEP can outpace increases in ER with greater precipitation, leading to an overall increase in NEE (Niu et al., 2008). Conversely, some findings have suggested that elevated GEP may be tempered by corresponding increases in ER, resulting in no net gain in NEE, despite increased precipitation (Risch and Frank, 2007). Thus, the magnitudes of the ecosystem C flux responses to changes in precipitation vary. To deepen our understanding of these effects and determine the pattern of C flux responses to both increased and decreased precipitation (linear or nonlinear), it is imperative to design experiments that would allow for simultaneous measurement of responses to both conditions within a single experimental framework.

Semiarid grasslands are highly sensitive to changes in precipitation (Liu et al., 2021; Post and Knapp, 2021). However, considerable uncertainty remains regarding the intricate ways in which precipitation shapes these ecosystems (Ahlström et al., 2015). Gaining insight into C cycling within semiarid grasslands as they respond to precipitation change can sharpen our predictive abilities regarding how grassland C cycles will respond to global climate change. This study focused on a typical semiarid grassland, the Songnen Meadow Steppe, using an *in situ* precipitation manipulation experiment (six precipitation gradients: P–70%, P–50%, P–30%, P+0%, P+30%, and P+50% relative to natural precipitation). During two consecutive growing seasons (2017 and 2018), we measured ecosystem C fluxes, plant biomass, and soil characteristics (SWC and soil temperature). This study aimed to answer the following questions: (1) How do precipitation changes affect ecosystem C fluxes? (2) Are ecosystem C fluxes equally sensitive to increased versus decreased precipitation, and are their responses symmetrical? (3) What are the underlying mechanisms by which precipitation changes regulate ecosystem C fluxes?

## 2 Materials and methods

### 2.1 Study site

The experimental site is located in the Songnen grassland ecosystem at the eastern end of the Eurasian Steppe in China (44°40′-44°44′N, 123°44′-123°47′E). This grassland has been fenced off for over 20 years, excluding grazing and other disturbances. The study area experiences distinct seasons: spring is dry and windy, summer is hot and rainy, autumn is mild with little rainfall, and winter is dry and cold, with significant differences in precipitation across the seasons. Interannual variability in precipitation is pronounced, ranging from 259 to 695 mm over the past few decades (1989–2018). Most precipitation occurs between June and August, accounting for approximately 70% of the annual total precipitation. The mean annual temperature in the region varies from 5.2 to 7.5°C (1989–2018) (Li et al., 2023). The study area is classified as a semiarid meadow steppe, with *Leymus chinensis* as the dominant plant species. Other prevalent species include *Hemarthria altissima* and *Phragmites australis* (Li et al., 2019). The soil in the study site is characterized by high pH and elevated salinity, with a soil organic C concentration of 0.9%, total nitrogen concentration of 0.08%, and phosphorus content of 0.02% (Yang et al., 2021).

### 2.2 Experimental design

In 2015, a 1 ha (100 m × 100 m) experimental plot was established in the study grassland, enclosed with a fence to isolate it from external disturbances. The fenced area is flat with relatively homogeneous vegetation, dominated by *L. chinensis*, which covers more than 85% of the area. There were no significant differences in plant community composition, aboveground biomass, or soil properties (total carbon, total nitrogen, total phosphorus, SWC, soil temperature, soil pH, and soil electrical conductivity) (Chai et al., 2022). Within the fenced grassland, four experimental blocks (30 m × 30 m) were established, with at least 3 m separating each block. In each block, six plots (3.5 m × 3.5 m) were designated, with a minimum buffer zone of 1 m between plots. To prevent lateral runoff between the plots and the surrounding area, each plot was enclosed by iron sheets (0.15 m aboveground, 0.5 m belowground). The six plots in each block were randomly assigned to one of six precipitation treatments: reduction in ambient precipitation by 70% (P–70%), 50% (P–50%), and 30% (P–30%); ambient precipitation (P+0%); and an increase in ambient precipitation by 30% (P+30%) and 50% (P+50%).

The experiment began in April 2016, with rainfall exclusion devices set up from April to October each year, followed by a controlled precipitation manipulation experiment. The rainfall exclusion devices consisted of metal frames and rain-sheltering panels. The metal frames measured 3.50 m in length and 3.62 m in width. To intercept rainfall, one side of the metal frame was 1.20 m high, while the other side was 2.14 m high, forming a 10° angle with the ground. This height allowed air circulation within the plots, minimizing the impact of the rainfall exclusion devices on the microclimate. The rain-sheltering panels were composed of V-shaped clear acrylic bands (3.7 m long, 0.33 m wide, 3 mm thick, with > 90% light transmittance) arranged in a grid on top of the metal frame. The proportion of area covered by the panels corresponded to the targeted reduction in precipitation. By installing 4, 6, or 8 rain-sheltering panels on the frame, we simulated reductions of 30%, 50%, and 70% of natural precipitation, respectively (**Supplementary Figure S1**). To avoid confounding effects from the rainfall exclusion devices, similar setups were installed for the control and increased precipitation treatments. Containers were placed next to each plot to collect the intercepted rainfall. After each precipitation event, the rainfall collected from the P–30% and P–50% treatments were immediately manually water the corresponding P+30% and P+50% plots within the same experimental block under the same precipitation rate. The rainfall collected from P+0%, P+30%, and P+50% plots were immediately manually water back the respective plots under the same precipitation rate. To avoid edge effects, plant and soil samples were collected only from the central area of each plot (2.5 m × 2.5 m) (Li et al., 2019).

### 2.3 Meteorological and soil environmental data collection

Meteorological data (precipitation and air temperature) were collected from a weather station (HOBO U30-NRC, Onset Computer Corporation, Bourne, MA, USA) located 15 km from the experimental site. The station continuously monitored natural precipitation and air temperature, recording data every 30 minutes. From May to September in both 2017 and 2018, SWC and soil temperature were measured monthly in all plots at depths of 0–15 cm. SWC was determined using the gravimetric method, with the following procedure: approximately 10 g of fresh soil from each sample was placed in pre-weighed, clean aluminum containers and dried at 105°C to a constant weight. The dry soil and container were then weighed to calculate SWC. Soil temperature was measured using a temperature probe (6000-09TC) connected to an infrared gas analyzer (LI-6400, LiCor Inc., Lincoln, NE, USA).

### 2.4 Measurement of aboveground net primary productivity

Aboveground net primary productivity (ANPP) was measured in August of 2017 and 2018. In each experimental plot, a 1 m × 0.5 m quadrat was randomly placed, and all living aboveground plant material within the quadrat was harvested. The collected plants were brought back to the laboratory, dried at 65°C to a constant weight, and then weighed. This value represents the aboveground biomass of the sampled quadrat. Since grassland plants die off each winter and regrow the following year, the aboveground biomass measured at this time also serves as an estimate of the ANPP for that year.

### 2.5 Measurement of ecosystem C fluxes

Ecosystem C fluxes were measured using a portable photosynthesis system (Li-6400, Li-Cor, Inc., Lincoln, NE, USA) in conjunction with the static chamber method. During the growing seasons of 2017 and 2018 (May to September), measurements were taken once per month, around mid-month, between 8:00 AM and 12:00 AM on clear, cloudless days. To ensure the airtightness of the photosynthesis chamber, a horizontal stainless steel frame (0.5 m × 0.5 m) was permanently installed in each plot at the start of the experiment, serving as the base for the chamber. When measuring ecosystem C fluxes, the photosynthesis system was connected to a transparent acrylic chamber (0.5 m × 0.5 m × 1 m), which was placed on the stainless steel frame, covering all the plants within the frame to maintain a sealed environment. Four small fans were installed at the top of the chamber and operated continuously during measurements to ensure uniform air circulation. Once the CO_2_ concentration inside the chamber stabilized, the photosynthesis system automatically recorded CO_2_ concentrations every 10 seconds for a duration of 2 minutes. The rate of change in CO_2_ concentration over time was used to calculate NEE. After this initial measurement, the chamber was lifted to allow the internal air to equilibrate with the outside atmosphere. Afterward, the chamber was placed back on the frame and covered with a lightproof cloth. Once the CO_2_ concentration stabilized, the photosynthesis system recorded the ER data. Gross ecosystem productivity was calculated as the difference between NEE and ER (GEP = – NEE + ER).

### 2.6 Data processing and statistical analysis

All statistical analyses were performed using R 3.6.0, with significance set at *P* < 0.05. First, linear mixed-effects models (LMMs) were used to analyze the effects of precipitation treatments on soil characteristics (SWC, temperature), ANPP, and ecosystem C fluxes (GEP, ER, and NEE). Subsequently, the interaction effects of precipitation treatments with measurement time and interannual variability were assessed using LMMs. The *lmer* function from the ‘*lme4*’ package was utilized to fit the LMMs, while Tukey tests were carried out using the *glht* function from the ‘*multcomp*’ package to compare the effects of altered precipitation. The sensitivity index of ecosystem C fluxes to precipitation change was expressed as the relative change in treated plots compared to control plots, calculated as follows:


(1) $$\text{Sensitivity}=\frac{\;(F_{ct}–F_{cc})/F_{cc}}{(SWC_{t}–SWC_{c})/SWC_{c}}$$

Here, *F_ct_* and *F_cc_* represent the ecosystem C fluxes in the precipitation treatment plots and control plots, respectively, while *SWC_t_* and *SWC_c_* denote the SWC in the precipitation treatment plots and control plots, respectively. A positive sensitivity indicates that the relative change in SWC causes a relative change in ecosystem C fluxes in the same direction. Conversely, a negative sensitivity indicates that the direction of the relative change in ecosystem C fluxes is opposite to that of the SWC.

For the relationship between SWC and various ecosystem C fluxes, both linear and nonlinear models were used for fitting. The linear model is as follows:


(2) $$F_{c}=\alpha SWC+\beta$$

The nonlinear model is as follows:


(3) $$F_{c}=\alpha \ln(SWC)+\beta$$

In the equation, *F_c_* represents the components of ecosystem C fluxes; *SWC* denotes SWC, *α* and *β* are the model fitting parameters. The final model selection was based on the Akaike Information Criterion (AIC).

Nonlinear regression analysis was employed to examine the relationships between various influencing factors and components of ecosystem C fluxes. Graphs were generated using SigmaPlot 12.5 (Systat Software Inc., San Jose, CA, USA). Structural Equation Modeling (SEM) was used to analyze the contributions of soil characteristics (moisture and temperature) and ANPP to ecosystem C fluxes (GEP, ER, and NEE). SEM allows for the testing of multivariate hypotheses, where some variables can simultaneously serve as both predictors and response variables (Grace, 2006). During the SEM construction process, the model fit was evaluated using the chi-square goodness-of-fit statistic and associated *P*-values. The model was analyzed using Amos 23.0 (Amos Development Corporation, Chicago, IL, USA).

## 3 Results

### 3.1 Abiotic and biotic factors

The magnitude and seasonal distribution of the precipitation events in 2017 and 2018 significantly differed (**Supplementary Figure S2**). Despite considerable interannual differences in precipitation, both years experienced dry conditions and limited rain in the spring, followed by wet and rainy conditions in the summer. During the growing seasons (May to September), the natural precipitation levels were 410.80 mm in 2017 and 312.80 mm in 2018 (**Supplementary Figure S2**). Precipitation treatments significantly changed the SWC, as it increased with increasing precipitation (*P* < 0.001, **Supplementary Figure S3**). For each year, across the treatments, the highest SWC was observed in the P+50% plots (2017: 21.19%; 2018: 19.69%), whereas the lowest was observed in the P–70% plots (2017: 10.84%; 2018: 5.84%). SWC showed significant interannual differences (*P* < 0.001), and there were significant interactions between different precipitation treatments and experimental years that affected SWC (*P* < 0.05, **Supplementary Figure S3**). In addition, although soil temperature during the growing season was inversely proportional to total precipitation, the impact of various precipitation treatments on soil temperature was not statistically significant (**Supplementary Figure S3**).

Precipitation treatments significantly affected ANPP (*P* < 0.001). In both 2017 and 2018, ANPP tended to increase proportionally to the extent of total precipitation (**Supplementary Figure S4**). In 2017, ANPP was significantly higher than that under the P+0% treatment, peaking at 375.70 ± 26.88 g m^-2^ under the P+50% treatment. Conversely, decreased precipitation significantly reduced ANPP, with the lowest value observed under the P–70% treatment (103.95 ± 14.62 g m^-2^). In 2018, although the increased precipitation improved ANPP compared with that under the P+0% treatment, the effect was not statistically significant. However, decreased precipitation significantly reduced ANPP, up to its lowest value under the P–70% treatment (92.55 ± 10.16 g m^-2^, **Supplementary Figure S4**).

### 3.2 Responses of ecosystem C fluxes to precipitation change

**Figure 1:**
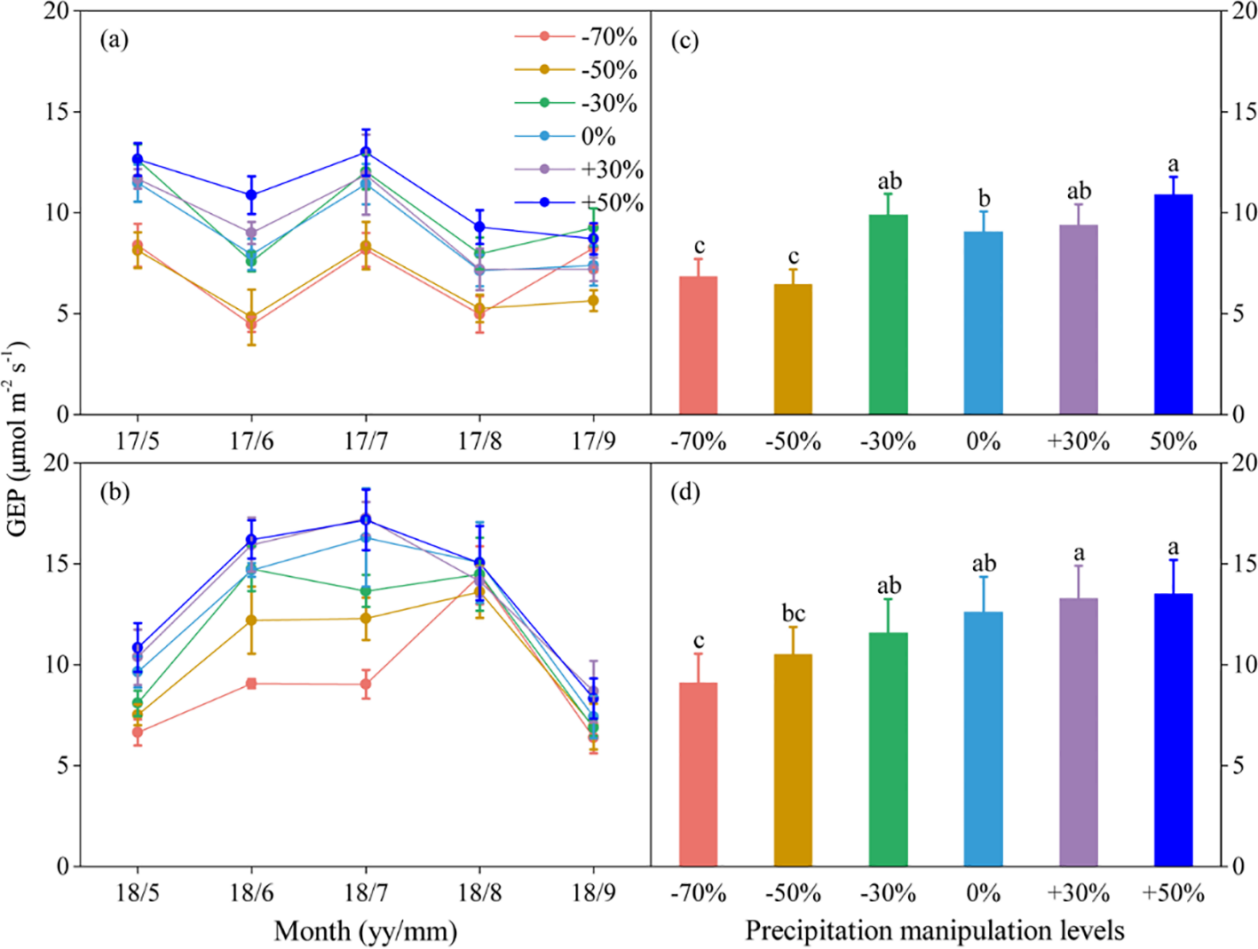
Seasonal dynamics **(A, B)** and means of gross ecosystem productivity (GEP) under different precipitation treatments **(C, D)** in 2017 and 2018. The right columns present seasonal mean GEP, and values are mean ± SE (n = 4). Different letters indicate significant differences between treatments in each experimental year at *P* < 0.05.

**Figure 2:**
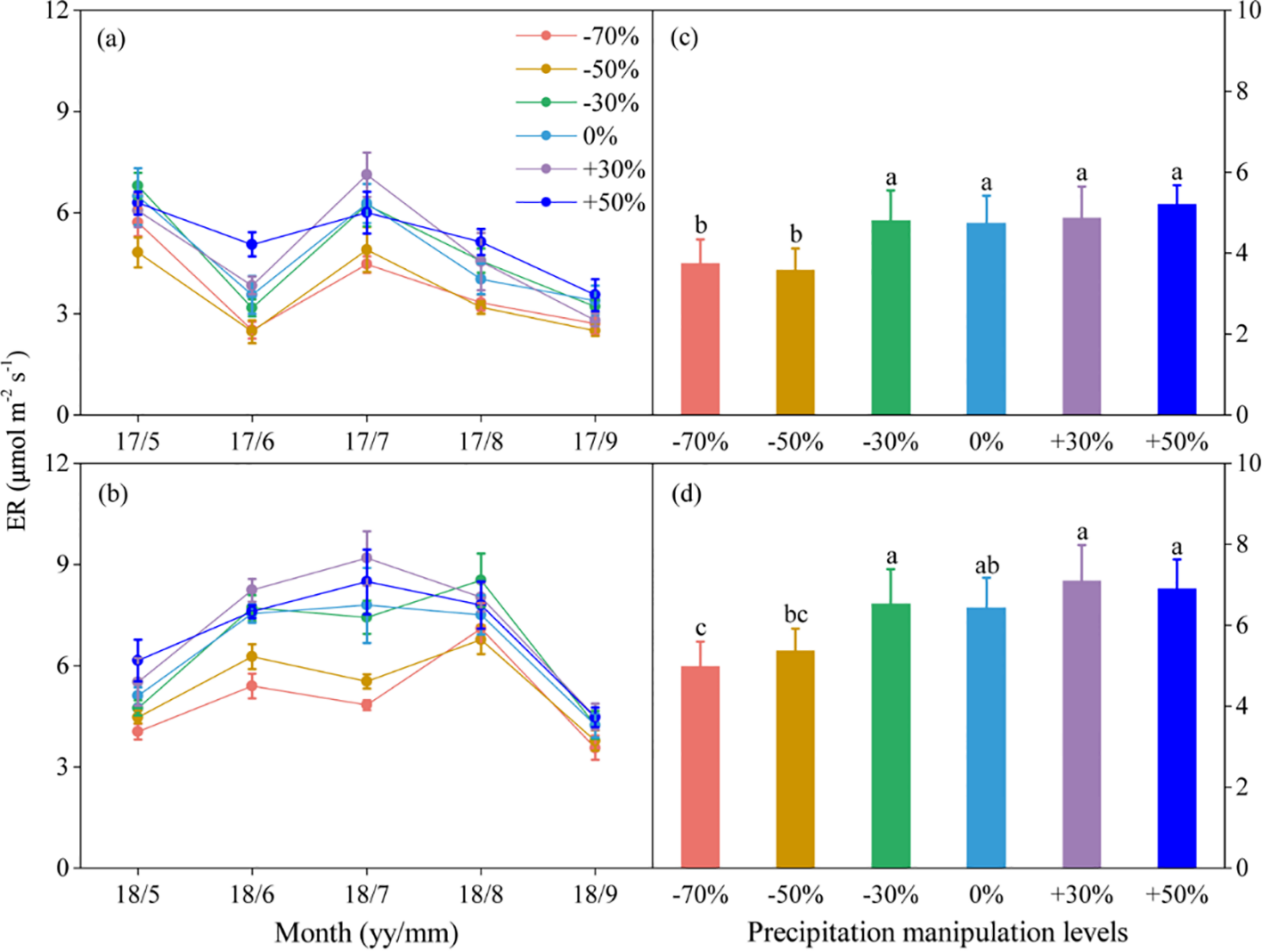
Seasonal dynamics **(A, B)** and means of ecosystem respiration (ER) under different precipitation treatments **(C, D)** in 2017 and 2018. The right columns present seasonal mean ER, and values are mean ± SE (n = 4). Different letters indicate significant differences between treatments in each experimental year at *P* < 0.05.

**Figure 3:**
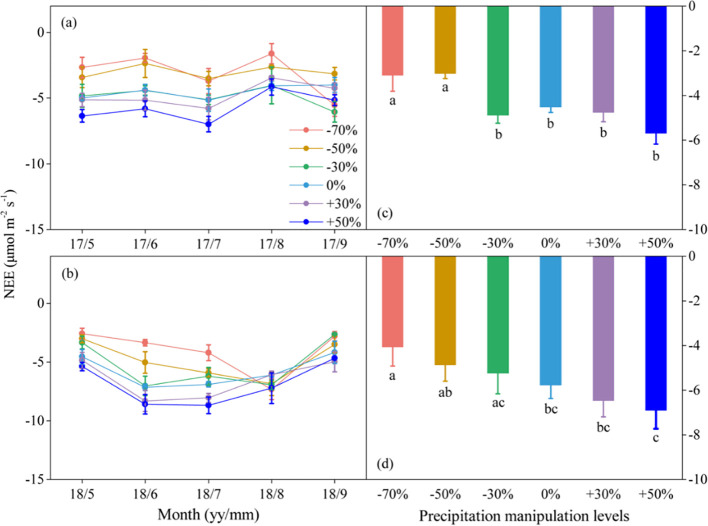
Seasonal dynamics **(A, B)** and means of net ecosystem CO_2_ exchange (NEE) under different precipitation treatments **(C, D)** in 2017 and 2018. The right columns present seasonal mean NEE, and values are mean ± SE (n = 4). Different letters indicate significant differences between treatments in each experimental year at *P* < 0.05.

Precipitation changes significantly affected ecosystem C fluxes (**Table 1**). On average, increased precipitation improved the GEP by 4.34% and 13.60% under the P+30% and P+50% treatments, respectively, whereas ER was increased by 6.47% and 8.49%, and NEE was increased by 8.66% and 22.84%, respectively (**Figures 1**–**3**). Conversely, decreased precipitation reduced GEP by 0.36%, 22.83%, and 26.19% under the P–30%, P–50%, and P–70% treatments, respectively, whereas ER decreased by 1.32%, 20.62%, and 21.81%, and NEE declined by 0.56%, 24.49%, and 30.59%, respectively (**Figures 1**–**3**). In 2017 and 2018, the temporal dynamics of GEP, ER, and NEE showed similar seasonal patterns, with peaks occurring during July and August, and a single-peaked seasonal pattern was observed during the 2018 growing season (**Figures 1**–**3**). After three years of precipitation manipulations, the ecosystem C fluxes (GEP, ER, and NEE) showed significant interannual differences (**Table 2**). Furthermore, significant correlations were observed between NEE and GEP, as well as ER, but the variations in NEE were more dependent on changes in GEP than in ER (*P* < 0.001, **Supplementary Figure S5**).

**Table 1:** The repeated measures analysis of variance for precipitation (P), measurement times (T) and their interactive effects on ecosystem CO_2_ fluxes in 2017 and 2018.

Variable	GEP	ER	NEE
2017			
P	**<0.001**	**<0.001**	**<0.001**
T	**<0.001**	**<0.001**	0.64
P × T	0.31	0.99	**<0.05**
2018			
P	**<0.01**	**<0.001**	**<0.001**
T	0.40	0.28	0.91
P × T	0.83	0.94	0.49

GEP, gross ecosystem productivity; ER, ecosystem respiration; NEE, net ecosystem CO_2_ exchange.

Bold values indicate *P* < 0.05.

**Table 2:** The repeated measures analysis of variance for precipitation (P), year and their interactive effects on ecosystem CO_2_ fluxes in 2017 and 2018.

Variable	GEP	ER	NEE
P	**<0.001**	**<0.001**	**<0.001**
Year	**<0.001**	**<0.001**	**<0.001**
P × Year	0.50	0.77	0.40

GEP, gross ecosystem productivity; ER, ecosystem respiration; NEE, net ecosystem CO_2_ exchange.

Bold values indicate *P* < 0.05.

### 3.3 Sensitivity of ecosystem C fluxes to precipitation change

**Figure 4:**
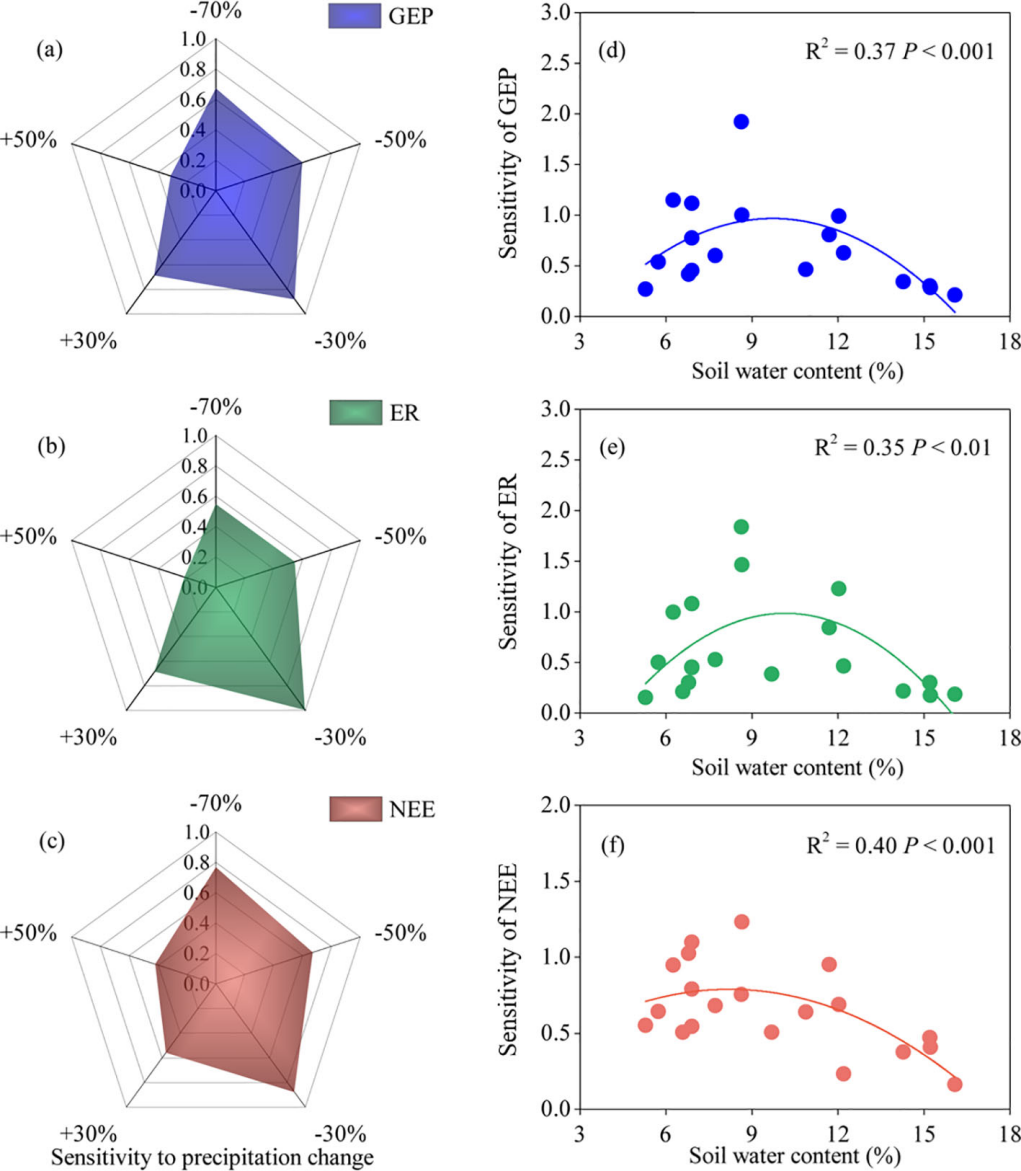
Sensitivity of ecosystem C fluxes to precipitation manipulation treatments. GEP, gross ecosystem productivity; ER, ecosystem respiration; NEE, net ecosystem CO_2_ exchange. The radar diagram shows the variation of sensitivity of ecosystem C fluxes under different precipitation treatments **(A–C)**, and the scatter diagram shows the variation trend of sensitivity of ecosystem C fluxes with soil water content **(D–F)**.

The ecosystem C fluxes (GEP, ER, and NEE) showed a nonlinear relationship with the gradient variation in SWC (**Supplementary Table S1**). We found that the sensitivity of ecosystem C fluxes to precipitation change was greater in the plots with low precipitation than in those with high precipitation, which led to a nonlinear response of ecosystem C fluxes to the precipitation gradient (**Figure 4**). Ecosystem C fluxes were most sensitive to the P−30% treatment among all low-precipitation conditions. Overall, under decreased precipitation treatments, NEE showed greater sensitivity to precipitation changes than GEP and ER (**Figure 4**). Conversely, under increased precipitation treatments, GEP was more sensitive to precipitation changes than ER and NEE (**Figure 4**). The sensitivity of ecosystem C fluxes to precipitation changes showed a significant quadratic regression relationship with SWC, indicating a threshold effect on the sensitivity of ecosystem C fluxes to precipitation change (**Figure 4**).

### 3.4 Pathways of precipitation change impact on ecosystem C fluxes

**Figure 5:**
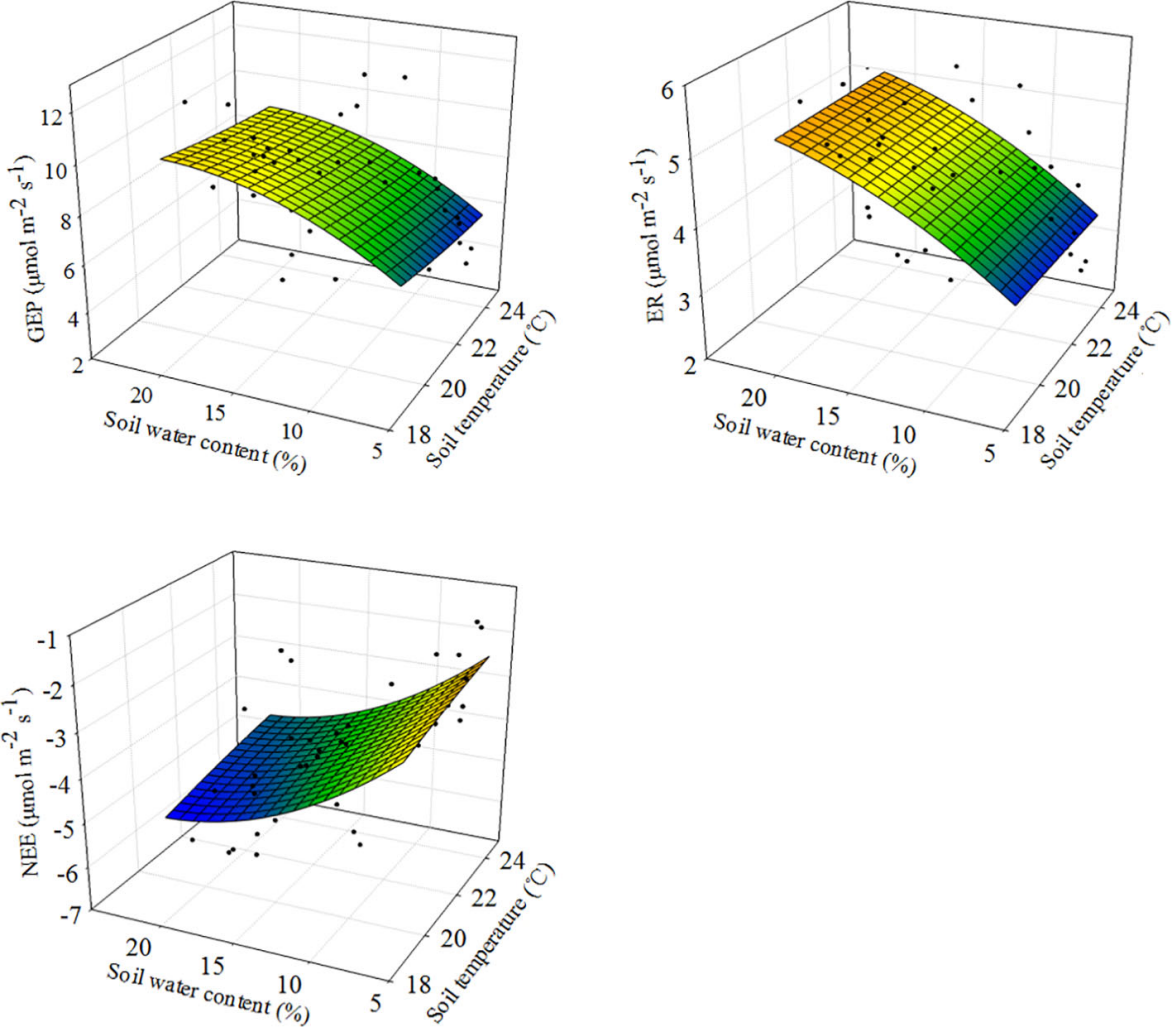
Response surfaces showing the relationships between soil water content and soil temperature versus ecosystem C fluxes across plots and years. GEP, gross ecosystem productivity; ER, ecosystem respiration; NEE, net ecosystem CO_2_ exchange. Modeled values (colored surfaces) are predictions from the models fitted with observations.

**Figure 6:**
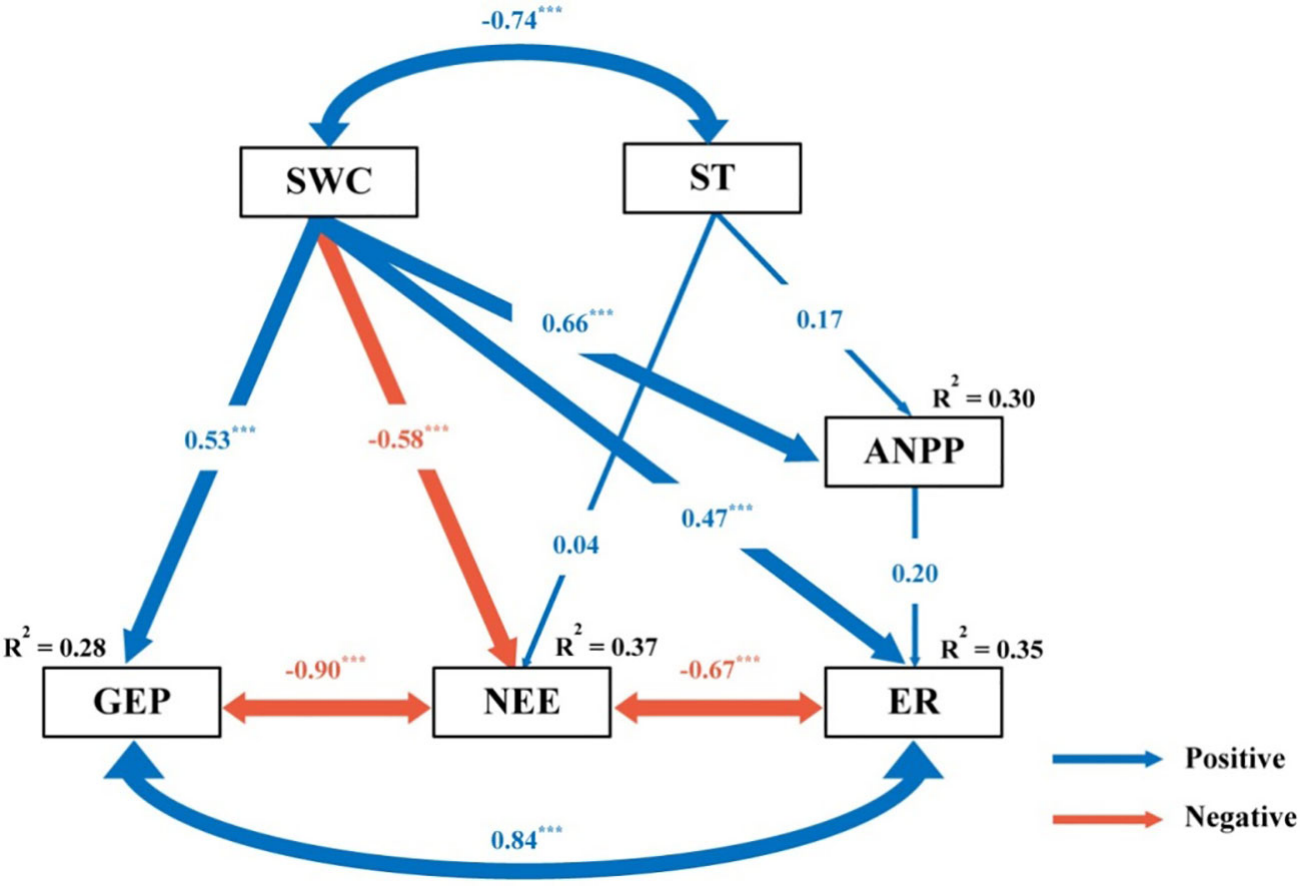
Results of structure equation model (SEM) analysis examining the effect of precipitation change on gross ecosystem productivity (GEP), ecosystem respiration (ER) and net ecosystem CO_2_ exchange (NEE), via pathways of soil water content (SWC), soil temperature (ST) and above-ground net primary productivity (ANPP). Blue and red arrows indicate positive and negative relationships, respectively. Arrow width is proportional to the strength of the relationship. The values adjacent to arrows are standardized path coefficients which reflect the effect size of the relationship. R^2^ values associated with variables indicate the proportion of variation explained by relationships with other variables. Significant level: ^***^ *P* < 0.001.

SWC was the primary factor driving changes in ecosystem C fluxes (**Figures 5**, **6**). Across all measurements, as SWC increased, ecosystem C fluxes also tended to increase non-linearly, in an exponential fashion (**Figure 5**; **Supplementary Table S1**). Moreover, the response surface indicated that the ecosystem C fluxes did not change significantly with variations in soil temperature (**Figure 5**). The SEM analysis revealed that ecosystem C fluxes responded differently to precipitation changes under the combined influence of environmental (SWC and ST) and biological factors (ANPP) (**Figure 6**). Although ANPP showed a significant correlation with ecosystem C fluxes, changes in GEP, ER, and NEE were primarily driven by the direct pathway through SWC (**Figure 6**; **Supplementary Figure S6**).

## 4 Discussion

### 4.1 Effects of precipitation changes on ecosystem C fluxes

Precipitation changes significantly alter ecosystem C fluxes (GEP, ER, and NEE), and with the duration of precipitation manipulation increased, the range of variation in ecosystem C fluxes affected by different precipitation treatments also increased (**Figures 1**–**3**). This phenomenon may be partly due to the lag effect of precipitation changes on SWC, which subsequently delayed their impact on ecosystem C fluxes. Numerous studies have confirmed the close relationship between SWC and components of ecosystem C fluxes (Zhang et al., 2017; Zhang et al., 2019). Consequently, the lag effect of SWC would inevitably influence ecosystem C fluxes responses to precipitation changes. Another contributing factor is the uneven magnitude and seasonal distribution of precipitation events. The dominant C_3_ plants have distinct water demand cycles in the Songnen grassland (Zhong et al., 2017), and ecosystem C fluxes are significantly correlated with precipitation during the early growing season (April to June), but not during other periods of the growing season (Wang et al., 2018). Compared with the conditions in 2018, the early growing season in 2017 was marked by drought (**Supplementary Figure S2**). Although sufficient rainfall occurred during the later stages of the growing season, *L. chinensis* (a C_3_ photosynthetic pathway species) grew slowly at high temperatures. Consequently, the uneven distribution of precipitation in 2017 reduced the effect of the precipitation gradient on grassland plots under different treatments.

Furthermore, we found that different ecosystem C flux components responded differently to precipitation changes (**Table 1**). The relationship between GEP and NEE (R² = 0.88) was considerably stronger than that between ER and NEE (R² = 0.70) (**Supplementary Figure S5**). This suggests that GEP had a stronger influence on changes in NEE than on changes in ER, which is consistent with previous findings (Zhang et al., 2017, 2019). This phenomenon likely occurs because precipitation change has a more pronounced effect on GEP than on ER (Zhang et al., 2017). GEP is intricately linked to plant growth, which is profoundly shaped by the variability in precipitation (Niu et al., 2008). In contrast, ER is influenced by a combination of environmental factors, such as temperature and precipitation (Quan et al., 2019).

### 4.2 Nonlinear response of ecosystem C fluxes to precipitation change

The difference in the sensitivity of ecosystem C fluxes to decreased and increased precipitation resulted in a nonlinear response to precipitation change (**Figure 4**). In this study, when precipitation decreased, the ecosystem C fluxes declined significantly, whereas increased precipitation left these fluxes largely unaffected (**Figures 1**–**3**). This indicated that the sensitivity of ecosystem C fluxes to decreased precipitation was greater than to increased precipitation. Ecosystem C fluxes were highly sensitive to decreased precipitation, which contrasted sharply with the more muted response to increased precipitation, leading to a nonlinear response of ecosystem C fluxes to precipitation change (**Figure 4**). Intriguingly, this sensitivity gradually decreased with increasing SWC, suggesting a threshold response of ecosystem C fluxes to precipitation change. These findings are consistent with previous reports that ecosystem C fluxes increase with SWC and peak at the optimal SWC (Jassal et al., 2008; Rojas-Robles et al., 2020).

The extreme drought condition intensified the nonlinear response of ecosystem C fluxes to the precipitation gradient. Although ecosystem C fluxes generally decreased with decreased precipitation compared with their values at the natural precipitation level (P+0%), significant decreases in GEP, ER, and NEE were only observed under extreme drought conditions (P–70%) (**Figures 1**–**3**). This further intensified the nonlinear response of ecosystem C fluxes to decreased precipitation, suggesting that future changes in precipitation, particularly extreme drought, would greatly hinder C fluxes. The nonlinear response of ecosystem C fluxes is mainly related to the nonlinear responses of SWC and ANPP to precipitation change (Wilcox et al., 2017). In this study, we found this to be the case, as both decreased and increased precipitation affected SWC and ANPP, but the sensitivity of these two variables to increased versus decreased precipitation was not consistent (**Supplementary Figures S3, S4**). Ultimately, this divergence led to a nonlinear response in ecosystem C fluxes, highlighting how even slight variations in SWC can amplify the nonlinear dynamics of C fluxes across the precipitation gradient.

### 4.3 Response mechanism of ecosystem C fluxes to precipitation change

Precipitation changes may profoundly influence ecosystem C fluxes by altering soil and plant properties (Arca et al., 2021; Wang et al., 2021a). Our results revealed that SWC was the main driver of changes in ecosystem C fluxes (**Figure 6**). Numerous researches have emphasized the crucial importance of soil water availability in the regulation of ecosystem C fluxes (Xia et al., 2009; Wang et al., 2021b). First, reduced SWC negatively affects plant growth by diminishing the leaf area and stomatal conductance, thereby limiting photosynthesis (Lavergne et al., 2020). Additionally, a decrease in SWC can disrupt plant CO_2_ uptake, impairing metabolic functions and limiting the dissolution of vital nutrients (Lawlor, 2002). However, diminished SWC also constrains CO_2_ release (Davidson et al., 2000). As moisture decreases, microbial activity declines, limiting the contact between microbes and available substrates, and consequently reducing the decomposition capacity of organic matter (Manzoni et al., 2012). Moreover, microbial utilization of soluble organic C and the activity of extracellular enzymes responsible for organic matter decomposition require liquid transport; thus, the lack of SWC hampers the microbial decomposition capacity (Grant and Rochette, 1994). In contrast, an increase in SWC has a revitalizing effect, fostering a positive response in ecosystem C fluxes.

Soil temperature is a pivotal driver of ecosystem C fluxes (Quan et al., 2019), however, in this study, soil temperature was not an important factor driving the changes in ecosystem C fluxes (**Figure 6**). This may be because the changes in precipitation did not significantly alter soil temperature (**Supplementary Figure S3**). Studies have shown that the range of soil temperature changes is relatively small and not sufficient to significantly impact C fluxes (Vleeshouwers and Verhagen, 2002). The response of the ecosystem to temperature changes usually requires a certain critical value before evident changes can be observed (Zhou et al., 2008). For example, an increase in soil temperature may promote the decomposition by microorganisms, but if the increase in temperature is relatively small, it may not be sufficient to lead to significant C release (Li et al., 2017). Precipitation plays a crucial role in directly influencing plant growth, which not only sustains plant cover, but also provides an essential substrate for C turnover, which is crucial for both C uptake and release (Zhang et al., 2017). Drought curtails plant productivity and photosynthesis, leading to a decline in ecosystem C fluxes (Zhang et al., 2019). Interestingly, in this study, although precipitation change significantly affected ANPP, SEM indicated that ANPP was not a primary determinant of ecosystem C fluxes, which is consistent with the findings of Zhang et al. (2017). Overall, in semiarid ecosystems, precipitation variation primarily controls ecosystem C fluxes through direct effects on SWC, whereas the roles of soil temperature and ANPP are less prominent.

## 5 Conclusion

This study revealed the nonlinear response of ecosystem C fluxes to precipitation gradients in a semiarid grassland ecosystem. In this environment, ecosystem C fluxes were highly sensitive to drought conditions, leading to a nonlinear response to precipitation change. Therefore, in future data integration and model predictions, the effects of decreased and increased precipitation on ecosystems should not be considered equivalent. Furthermore, changes in SWC, driven by precipitation variability play a critical role in regulating ecosystem C fluxes. This also implies that SWC plays a pivotal role in shaping ecosystem functions. Our findings highlight the nonlinear response of ecosystem C fluxes to increasing and decreasing precipitation. The ecosystem’s response to changes in precipitation suggests that ecosystems may show a broad range of nonlinear responses to global climate changes, including rising temperatures, increased CO_2_ concentrations, and nitrogen deposition. Therefore, future studies examining the effects of global climate change on ecosystems should incorporate multi-gradient experiments to provide a reliable data foundation for assessing ecosystem C cycling. Furthermore, future ecological models should incorporate the response patterns of various ecosystem components to precipitation changes, enriching our capacity to understand and predict the intricate responses and feedbacks of ecosystem C cycling to global climate change.

## Data Availability

The raw data supporting the conclusions of this article will be made available by the authors, without undue reservation.
