# Supplementary material for: Nonlinear responses of ecosystem carbon fluxes to precipitation change in a semiarid grassland
Source: Front Plant Sci. 2025 Feb 6;16:1519879. doi: 10.3389/fpls.2025.1519879 (PMC11840572; doi:10.3389/fpls.2025.1519879)
Supplement: Supplementary file 1 [file DataSheet1.doc]

**Nonlinear responses of ecosystem carbon fluxes to precipitation change in a semiarid grassland**

**
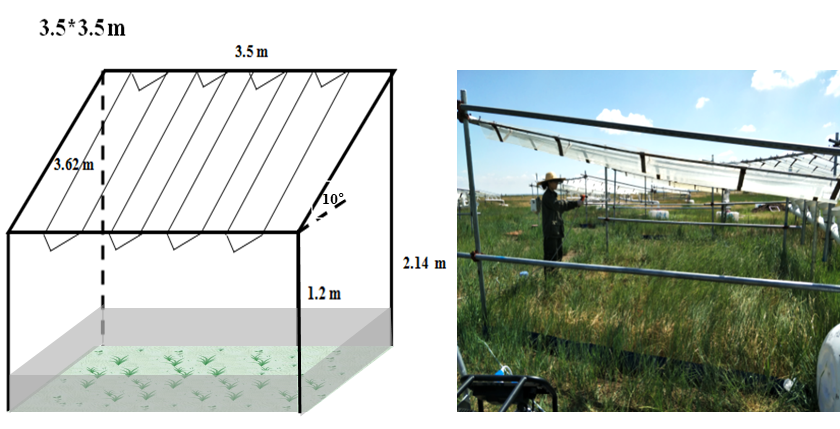
**

**Supplementary Figure 1.** The design of rainout shelter (left) and a picture of precipitation treatment (right).


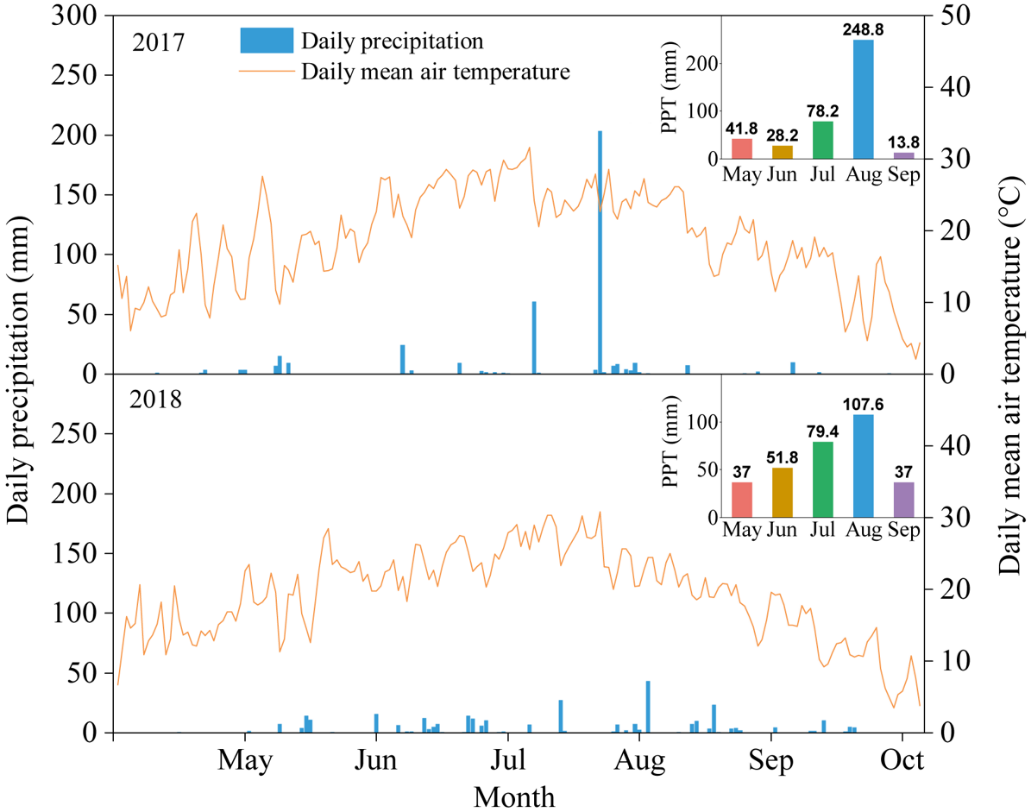


**Supplementary Figure 2.** Daily precipitation (mm) and daily mean air temperature (°C) from April to October in 2017 and 2018. Inset shows the month precipitation amounts during the growing season.


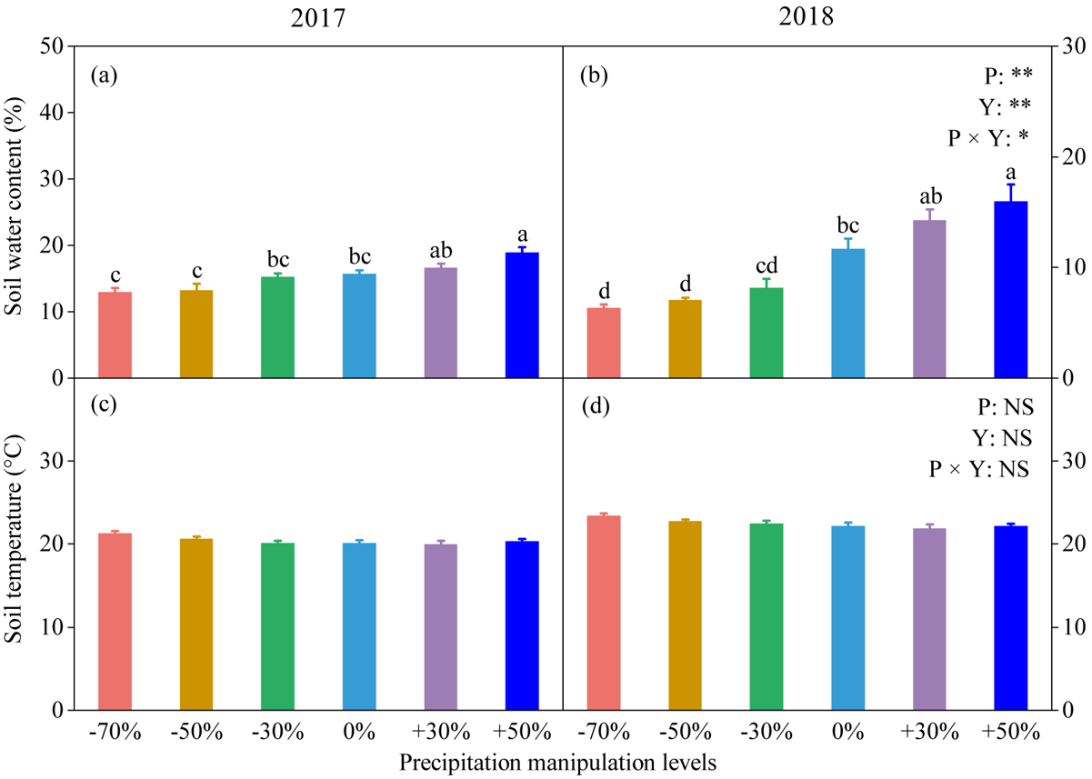


**Supplementary Figure 3.** Variation in soil water content (a, b) and soil temperature (c, d) under different precipitation treatments in 2017 and 2018. P: precipitation treatment; Y: year. The values are the means of multiple measurements from May to September. Error bars represent SE. Different letters indicate significant differences between precipitation treatments in each year. Level of significance: ^*^, *P* < 0.05; and ^**^, *P* < 0.001; NS, *P* > 0.05.


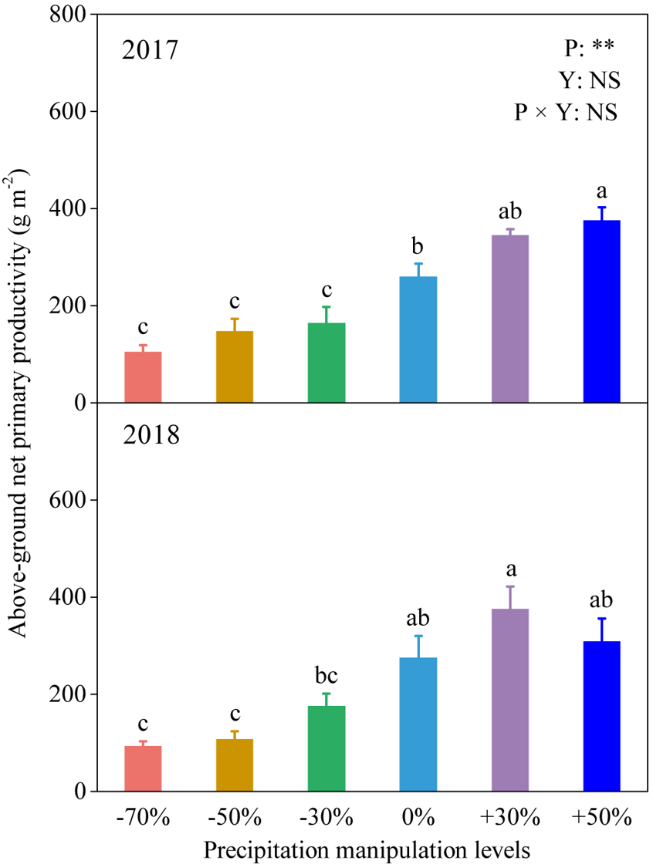


**Supplementary Figure 4.** Variation in above-ground net primary productivity under different precipitation treatments in 2017 and 2018. P: precipitation treatment; Y: year. Values are means ± 1 SE of four replicates. Different letters indicate significant differences between precipitation treatments in each year. Level of significance: ^*^, *P* < 0.05; and ^**^, *P* < 0.001; NS, *P* > 0.05.


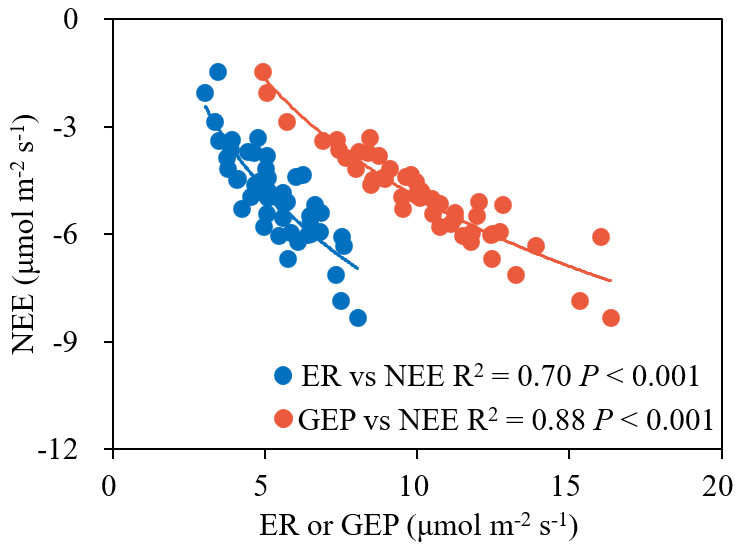


**Supplementary Figure 5.** Dependence of net ecosystem CO_2_ exchange (NEE) on gross ecosystem productivity (GEP) or ecosystem respiration (ER) across all experimental plots. Each data point represents a growing-season average of each plot over the two seasons.


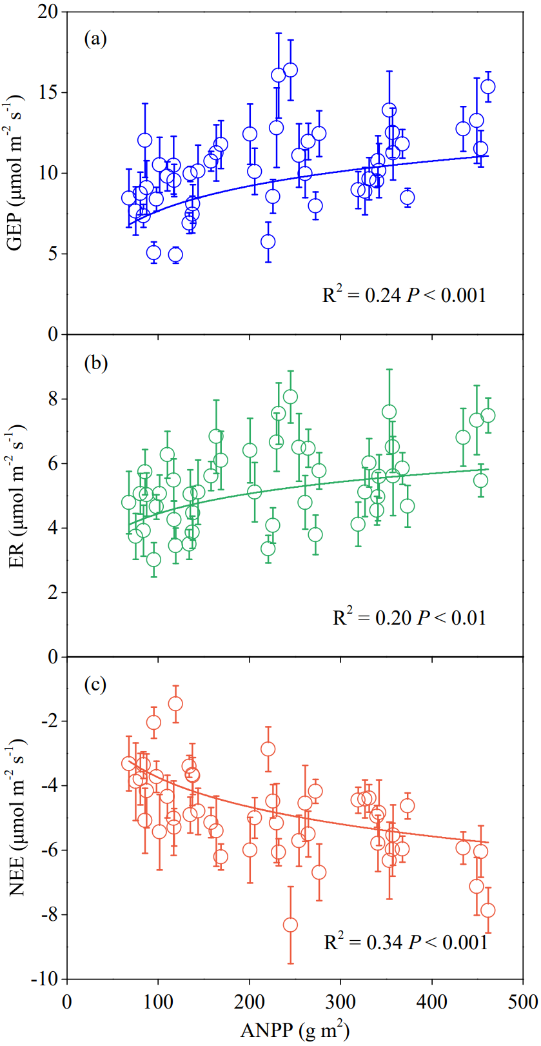


**Supplementary Figure 6.** Relationships between ecosystem C fluxes and above-ground net primary productivity (ANPP) across seasons and plots. GEP, gross ecosystem productivity (a); ER, ecosystem respiration (b); NEE, net ecosystem CO_2_ exchange (c).

Supplementary Table 1. AIC and R^2^ of the linear and nonlinear models between annual mean ecosystem C fluxes and soil water content. GEP, gross ecosystem productivity; ER, ecosystem respiration; NEE, net ecosystem CO_2_ exchange.

|  | AIC | | R^2^ | |
| --- | --- | --- | --- | --- |
|  | Linear model | Nonlinear model | Linear model | Nonlinear model |
| GEP | 209.86 | 208.52 | 0.35 | 0.37 |
| ER | 136.09 | 135.79 | 0.39 | 0.40 |
| NEE | 140.05 | 138.38 | 0.45 | 0.47 |
